# Meeting psychosocial needs for persons with dementia in home care services – a qualitative study of different perceptions and practices among health care providers

**DOI:** 10.1186/s12877-017-0612-3

**Published:** 2017-09-11

**Authors:** Anette Hansen, Solveig Hauge, Ådel Bergland

**Affiliations:** 1 University College of Southeast Norway, Faculty of Health and Social Sciences and Centre for Care Research, Postbox 235, 3603 Kongsberg, Norway; 2 Department of Nursing Science and Lovisenberg Diaconal University College, Institute of Health and Society, University of Oslo, Lovisenberggaten 15b, 0456 Oslo, Norway

**Keywords:** Home care services, Dementia care, Psychosocial, Holistic care, Health services research, Focus group, Qualitative research

## Abstract

**Background:**

The majority of persons with dementia are home-dwelling. To enable these persons to stay in their own homes as long as possible, a holistic, individual and flexible care is recommended. Despite a requirement for meeting psychological, social and physical needs, home care services seem to focus on patients’ physical needs. Accordingly, the aim of this study was to explore how the psychosocial needs of home-dwelling, older persons with dementia were perceived, emphasized and met by home care services.

**Methods:**

A descriptive, qualitative approach was used. Data were collected through semi-structured focus group interviews with 24 health care providers in home care services from four municipalities. Data were analysed using systematic text condensation.

**Results:**

This study showed major differences in how health care providers perceived the psychosocial needs of older home-dwelling persons with dementia and how they perceived their responsibilities for meeting those psychosocial needs. The differences in the health care providers’ perceptions seemed to significantly influence the provided care. Three co-existing logics of care were identified: the physical need-oriented logic, the renouncement logic and the integrated logic.

**Conclusions:**

The differences in how health care providers perceived the psychosocial needs of persons with dementia and their responsibilities for meeting those needs, influenced how the psychosocial needs were met. These differences indicates a need for a clarification of how psychosocial needs should be conceptualized and who should be responsible for meeting these needs. Further, increased competence and increased consciousness of psychosocial needs and how those needs can be met, are essential for delivering high-quality holistic care that enables persons with dementia to live in their own home for as long as possible.

**Electronic supplementary material:**

The online version of this article (10.1186/s12877-017-0612-3) contains supplementary material, which is available to authorized users.

## Background

Due to the increasing number of persons with dementia (PWDs), the World Health Organisation (WHO) recommends that governments declare dementia a public health priority [1]. Worldwide, the majority of PWDs are home-dwelling [2, 3]. This is also the case in Norway where over 50% of the 78,000 PWDs are living in their own homes [4], many of these live alone. Because most PWDs want to live in their own homes and the provision of home care services is more cost-efficient than institutionalized care, Norway as several other Western countries, aim to offer holistic, customized and individualized home care services to safeguard the needs of PWDs and their families [1, 2, 5].

Taking care of psychosocial needs is viewed as a central component of high-quality dementia care [6, 7], in which tailored and individual care beyond meeting physical needs, such as nutrition, medication, personal hygiene and domestic chores, is emphasized [8]. As PWDs are at risk of being isolated and lonely [9], social contact, confident relationship, and meaningful and varied activities are factors that must be emphasized to increase the quality of life [5, 10] and well-being of PWDs [11]. Impaired cognition and neuropsychiatric symptoms are common among PWDs and have a negative effect on quality of life [12]. Depression, anxiety, apathy and irritability are the most common neuropsychiatric symptoms of dementia [12]. Because the use of psychotropic drugs may have unpredictable effects and potential risks [12], nonpharmacological interventions have an important role in dementia care. Research concerning interventions to enhance psychological and social health has been addressed in various ways from cognitive therapy [13] and social support groups [14] to the use of reminiscence [15], music [16] and animals [17]. Despite the growing number of research on PWDs’ psychosocial health, needs and interventions [18–21], the majority of this research has been conducted within an institutional context, such as in nursing homes [6, 22]. These findings may not automatically be applicable to a home care setting because the needs of PWDs, the available health care providers (HCPs) and the possibility of implementing interventions may vary between the two settings. Research undertaken in home care services related to the psychosocial needs of PWDs often tends to focus on the family caregivers’ psychosocial health and needs [6], such as the burden of care [23, 24] or different dyadic interventions for PWDs and their family caregivers [6, 25, 26]. Indeed, the family caregivers play an important role in caring for PWDs, thus, caring and support for the family caregivers are crucial [26].

The shift from institutional care towards home care service highlights the focus on individual tailored care in the home care services [27]. High-quality home care services should be adjusted to the specific needs of PWDs such that they experience respect, predictability and security in relation to the care provided [2, 7, 28]. Turjamaa et al. [27] advocate that home care services must take into account the significance of the individual’s psychosocial needs, such as social contact and meaningful activities, as the receivers of home care services often show cognitive, psychological, social and functional decline [27]. Furthermore, the home care services must be flexible [2], person-centered [5, 7, 29] and provided by known persons with sufficient time [30]. In contrast to this ideal, home care services are often characterized by having many employees and a shortage of time [27, 31]. According to Kitwood [28], caring for PWDs generates specific organizational issues. The best care is given when the providers have high awareness, knowledge, sufficient resources and freedom to make independent choices based on the specific situations and the individuality of the PWD [28]. Creating a trusting relationship, and having good knowledge of the individual PWD’s background, situation, resources and needs may be challenging to achieve when many different, unknown or unqualified HCPs are delivering the care [32]. A lack of time may further reduce the ability to adjust care in a flexible manner to respond to the individual PWD’s actual and often changing needs [2]. Despite a requirement stating that cultural, social, and physical activities and thriving should be central elements of the healthcare services, there is evidence that these factors are often given low priority [5, 33]. Generally, research indicates that healthcare services have a greater focus on older patients’ physical needs than their psychosocial needs [33–35].

A number of studies have been conducted on the different aspects of home care services, such as the organization of the service [36, 37], the quality of service [38], the assessment of needs [39, 40] and the delivery of care [41, 42]. However, few studies have focused on home care services to specific groups of recipients, such as PWDs [43]. The limited number of studies that focus on the needs of PWDs who are receiving home care services, have primarily focused on needs in general, and not on specific psychosocial needs [44, 45]. Furthermore, there is scarce knowledge about the perspectives of HCPs’ regarding the needs of home-dwelling PWDs [44, 46], and to our knowledge, there are no studies specifically related to HCPs’ perceptions of the psychosocial needs of home-dwelling PWDs and how these perceptions may influence the care provision. To address this issue, the aim of this study was to explore how the psychosocial needs of home-dwelling, older PWDs were perceived, emphasized and met by home care services, based on descriptions from the HCPs.

## Methods

### Design

A descriptive qualitative design was used to develop knowledge and understanding [47].

### Setting

Each of the municipalities in Norway is responsible for providing healthcare services of good quality to its citizens in accordance with their necessary health care needs [48]. In large- and medium-sized Norwegian municipalities a purchaser-provider split model is often used to allocate and provide healthcare services (e.g., home care services and day care centres). The allocation and provision of healthcare services are separated in two different units: a purchaser unit and a provider unit. Applications for healthcare services are sent to the purchaser unit and the purchasers assess the patients’ needs and allocate services accordingly. The purchasers form an administrative decision describing the content and scope of the service [49]. The providers (e.g. registered nurses and assistant nurses in home care services) deliver services based on the administrative decisions. Norwegian municipalities have implemented the purchaser-provider split model to various degrees that have been adjusted to fit different local contexts [50]. In the present study, the allocation and provision of services were separated, and the HCPs were anticipated to provide services based on administrative decisions.

### Recruitment and participants

Invitation to participate in the study was sent to municipalities located in Eastern Norway. The first four municipalities, in which the leaders of the healthcare services responded positively, were included. Participants for the focus group interviews were recruited by the leaders of the home care service units, one group from each municipality. To gain a trustworthy view of the home care services, the two groups of HCPs that usually work within the services were asked to participate in the focus group interviews. The focus groups comprised of five to eight participants with 24 participants in total, including ten registered nurses and 14 assistant nurses, the latter with more than 6 months of healthcare education. The participants’ work experience in healthcare services ranged from four to 42 years, and all of the participants were female. In the following section, the participants are referred to as HCPs.

### Data collection

Semi-structured focus group interviews were used to explore how psychosocial needs were perceived, assessed and met by the HCPs. An interview guide with relatively open questions was used [51]. Examples included the following: “How do you understand psychosocial needs?”, “How do you meet PWDs’ psychosocial needs?” and “What do you do if you discover needs that are not described in the administrative decision?” (For complete interview guide se Additional file 1). The focus group interviews were conducted from a descriptive perspective, where the HCPs shared and discussed their perceptions and experiences and responded to each other’s statements and reflections. By discussing and sharing rich and nuanced descriptions, the HCPs also stimulated each other to consider new viewpoints concerning the topic [51]. The first author (AH) conducted the focus group interviews. The second author attended the first focus group interview as an assistant- moderator [51]. A brief note containing “main experiences” and comments that could improve further moderating, was completed immediately after each interview [51]. The focus group interviews were audio- recorded and lasted 90–120 min. Except from one interview, all the focus group interviews were held at the HCPs’ workplace at their request. Data were collected between September 2012 and June 2013.

### Data analysis

The audio-recorded focus group interviews were transcribed verbatim by the first author and listened to several times to ensure accuracy between the audio recordings and the transcripts [52]. The data- analysis was inspired by Malterud’s systematic text condensation [53]. The analysis process was conducted in four steps. In the first step, all interviews were read to gain an overall impression and identify preliminary themes. Examples of preliminary themes were ‘loneliness’, ‘holistic care’ and ‘shared responsibility’. In the second step, meaning units, related to the preliminary themes, that represented different aspects of HCPs’ descriptions concerning PWDs’ psychosocial needs were identified and coded under different headings. NVivo-10 software [54] was used in this second step to facilitate a systematic organization of the data. In the third step, each code group and subgroup was condensed and abstracted. Finally, the abstracted contents of the condensates were synthesized. The major findings were developed as a result of further interpretations of the synthesized condensates. To ensure that the synthesized statements reflected the wholeness of the original context, these statements were compared with the original data, both by reading the transcripts and listening to the audio-tape [53]. The first author held primary responsibility for conducting the analysis. However, all three authors had regular meetings throughout the entire analysis process during each stage. Development of themes, codes and code groups were discussed and refined until a consensus was reached. This cooperation between the three authors was important to secure each stage in the analysis process [53].

## Results

The analysis process identified differences in how the HCPs described psychosocial needs in PWDs, how they perceived their responsibility to meet these needs, and finally, how their perceptions influenced the care provided. In the following sections, we describe these differences according to three logics: the physical need-oriented logic, the renouncement logic and the integrated logic. Due to small overlaps and nuances, the three logics cannot be viewed strictly isolated. Both nurses and assistant nurses from all four municipalities were represented in all three logics.

### The physical need-oriented logic

In the physical need-oriented logic, physical and psychosocial needs were seen separately, and only physical needs were considered as basic needs, which mainly included help with personal hygiene, meals and medications. Psychosocial needs were restricted to needs for social stimuli, such as being with others, and needs for physical safety, such as a stove guard and a municipal assistance alarm.

Meeting PWDs’ psychosocial needs was considered a responsibility of the relatives or an issue that could be addressed outside the PWD’s home, for example in a day care centre. Assessing and meeting psychosocial needs were not seen as within the HCPs’ area of responsibility: *“... we are only a tool, we shall help them with what they need, not manage to do ... it’s the physical part we are sent out to, that’s our job”*.

Another characteristic of this logic was to keep strictly to the administrative decision and not provide care that was not allocated. Due to this mindset, the focus was task-oriented, and the care provided was described as being inflexible and uniform, as this usually characterizes administrative decisions. Consequently, care was provided in accordance with the minimum requirement because psychosocial needs seldom were described in the administrative decisions. The PWDs’ needs had to be adjusted to fit the HCPs’ time schedule and routines, and individual needs were seen as difficult to meet.

In this logic, sitting down and “just talking” with PWDs was not perceived as giving care. Additionally, meeting psychosocial needs was not considered as part of the HCPs’ job. PWDs who expressed loneliness and a need for social contact were usually met with boundaries: *“... we are not companion ladies ... it is not our job to sit down and talk with them”.* The need for social contact was further described as insatiable, based on the PWDs’ memory problems: *“They forget once we have been there, they forget that we have been there, that’s why they feel so lonely”*. The economy of the municipalities was also an argument for not meeting psychosocial needs. The resources were perceived allocated only to meet physical needs. If the HCPs should fulfil all PWDs’ psychosocial needs the home care services would collapse. This reasoning, mainly focusing on fulfilling physical needs described in the administrative decisions, and not exceeding time or financial limits, seemed to lead to a task-oriented way of performing care with little room for flexibility or professional judgments. Within this logic, assessing or meeting psychosocial needs seemed not to be perceived as within the HCPs responsibility.

### The renouncement logic

In this logic psychosocial needs were considered as basic needs, however, the physical needs were perceived more essential and had to be prioritized: *“It is the physical needs that comes first. There is a lack of focus on the psychosocial health and needs, and we should probably be better at emphasizing those needs, but the focus is on the physical. It’s about our understanding of the human and our view of human life”.* Assessing and caring for psychosocial needs was perceived as important, however, physical needs were considered most significant. The assessment and accommodation of psychosocial needs were described as more random and unconscious. Primary psychosocial needs were seen as a need of social contact, having a meaningful life and feeling socially and physically secure. Needs that arose from unrest, anxiety or depression seemed to be observed, but emphasizing and meeting these needs were not prioritized within this logic.

The HCPs claimed that many PWDs had limited possibilities to communicate about and fulfil their psychosocial needs. In contrast to the physical need-oriented logic, the HCPs in the renouncement logic perceived having a certain degree of responsibility for meeting psychosocial needs. However, one main characteristic of this logic was that the HCPs observed and assessed the PWDs’ psychosocial needs and suggested interventions, but they transferred the fulfilment of the psychosocial care to the relatives, e.g., if a HCP observed signs of sadness or loneliness in a PWD, the relatives were contacted to accommodate the necessary needs. In addition to relatives, the HCPs viewed day care centres and volunteer organizations as important contributors of fulfilling the PWDs’ psychosocial needs. Despite this view, the HCPs seldom contacted volunteers because they claimed that they had little knowledge of which volunteer organizations to contact and what the different organizations could offer.

Limited competence and consciousness regarding psychosocial health and needs were described to impede the care: *“I wish we could follow them to activities, identify their network… but we do not have sufficient time, knowledge or awareness concerning psychosocial health and needs. We should ask ourselves, what are psychosocial needs, and what are we doing to meet them?”* Despite the HCPs’ descriptions of insufficient competence and awareness to fulfil psychosocial needs, our data revealed a view focusing on “being within the moment”, as being unfocused or hurrying could increase insecurity and unrest in PWDs. In line with this view, care provided in a calm and friendly manner was emphasized. This logic emphasized conversations with the PWDs, although the conversations were often characterized as random, without any conscious goal or content. The conversations were also seldom conducted without simultaneously performing practical tasks. Because many PWDs may have a reduced ability to perceive oral communication, one HCP questioned the quality of these types of conversations: *“The quality of the conversation that we have while we stand with our backs to the patient, and butter the bread, no eye contact, is probably not a good conversation”*.

Caring for PWDs was described as time-consuming. Insufficient time was perceived preventing the HCPs from performing care related to psychosocial needs. One of the HCP explained the following: *“I would like to meet them on their loneliness and sadness, but I can’t, I can’t open too many things, because I have to leave them. I have to put a lid on it, because I have to go to the next patient”*. The focus on restricted time and a feeling of limited competence in meeting psychosocial needs seemed to hamper finding flexible solutions, also resulting in a feeling of insufficiency among the HCPs. The HCPs’ perceptions of lack of competence, consciousness and time seem to prevent them from meeting the psychosocial needs of the PWDs, and a transference of responsibility to fulfil these needs subsequently occurs.

### The integrated logic

In the third logic, the integrated logic, psychosocial needs were described as important basic needs, in line with physical needs: *“We must also meet the psychosocial needs, we have to see to all human needs. Actually, the psychosocial needs are very important”.* Psychosocial and physical health were considered to have a great influence on each other. Consequently, physical and psychosocial needs were not seen as separate, but rather as needs that influenced each other and often could be addressed simultaneously. Unmet psychosocial needs may lead to reduced physical health; for instance, an unfulfilled need for social contact could lead to reduced appetite and malnutrition. Care that leads to a meaningful daily life that encompasses mental and emotional factors was substantial in this logic. The PWDs’ social environment was described as significant for thriving as well as for maintaining cognitive and mental functions. Likewise, a trusted relationship and a good conversation were seen as valuable for maintaining identity and the person’s sense of self. Emphasizing security, continuity and predictability in the care provided would not solely enhance psychosocial health but also would make it easier to conduct practical tasks, such as assistance with bathing. Furthermore, good cooperation with relatives and volunteers was emphasized, especially concerning the fulfilment of social, cultural and spiritual needs.

In this logic, assessing and providing care related to psychosocial needs was a conscious, natural and integral part of the professional practice. As PWDs were not always able to describe or take care of these needs themselves, the HCPs’ perceived their responsibility to include more than physical needs. Despite a lack of time, a conscious utilization and effort to operate freely within the limited timeframe was apparent. An additional characteristic of this logic was that the flexible and individual care provided went beyond what was stated in the administrative decisions. This way of performing care was based on an understanding that the HCPs’ knowledge of dementia together with their knowledge of the PWD’s individual needs affected the provision of care, e.g., what the PWD perceived as meaningful had a great influence on how care was adapted and provided. For instance, if a PWD expressed loneliness or sadness, the HCP would adjust the time schedule so that she had the opportunity to sit down and talk. A good relationship between the HCPs and the PWDs was described as essential for increasing well-being and reducing anxiety and depression. Due to PWDs’ impaired cognition helping to increase understanding and meaning in everyday life situations became important. The HCPs explained this understanding by describing what care, based on this logic, could result in: *“… they become more confident, more open, dare to show more face, drop these protection mechanisms”.* By knowing the PWDs well, it was also easier to see their resources and facilitate for participation.

Another essential characteristic of this logic was that conversations were viewed as an important tool that had to be used purposely to identify as well as meet psychosocial needs. By identifying topics that the PWDs could manage to talk about, this could increase their sense of coping and contribute to strengthen the PWDs’ identity: *“It’s all about making good conversations, it is fantastic when you manage, you see it does something to them, the joy, the twinkle in their eyes, when they find their way back to themselves”.* In this logic, the conversations, performed while conducting practical tasks such as assisting medications or domestic work, were seen as important for meeting PWDs’ need for social contact and pleasure. However, the purpose of the conversation was extended further, it was used as a tool for assessing loneliness, anxiety or depression. If the PWDs’ needs went beyond what could be met during the conversation conducted while performing practical tasks, the HCPs saw it as their responsibility to prioritize extra time to talk: *“I take the time to sit down with them. Sit down to talk about the loneliness, the anxiety, assess the reason. Is there something we could do or do we have to contact a specialist? Communication is very important. I think they really appreciate when I sit down and just listen or talk, showing that I care”.*

## Discussion

The main findings in this study showed major differences in how HCPs perceived the psychosocial needs of home-dwelling PWDs and their responsibilities for meeting those needs. These differences seemed to influence the provided care, and are described in three co-existing logics; the physical need-oriented logic, the renouncement logic and the integrated logic.

The present study indicates that psychosocial needs are prioritized at a lower level than physical needs in home care services. This finding is supported by previous research on prioritization in home care services [33, 34]; however, these studies were not dementia-specific. The emphasis on physical needs can be seen in connection with research describing how psychosocial and spiritual needs tend not to be assessed as part of the services provided by home care services [33]. The latter is only partly in line with our results. Within the physical need-oriented logic this was the case; however, in the renouncement logic and the integrated logic, psychosocial needs were acknowledged as within the area of responsibility of home care services and were thus needs to which the HCPs had to relate.

How HCPs view their role in fulfilling PWDs’ psychosocial needs seems to be related to how HCPs perceive psychosocial needs. By viewing psychosocial needs as basic needs, needs for esteem, respect, love, safety and belonging are factors included as basic care [55]. Similar to the HCPs in the integrated logic, Edvardsson et al. [7] stated that psychological needs are basic needs equivalent to physical needs. Delivering services emphasizing psychosocial needs, that are equivalent to physical needs is important, especially to PWDs as they often have reduced opportunities to satisfy their own psychosocial needs, such as maintaining social contact [7]. When HCPs in the physical need-oriented logic describe “just sitting down to talk”, as not doing anything and consider meeting psychosocial needs not to be an aspect of their job, this is not an uncommon perspective; indeed, this view was reported in an earlier study [30]. It is interesting to see this in the context where central authorities advocates that PWDs should stay as long as possible in their own home and receive individually tailored services that also meet psychological, social and spiritual needs [5]. Additionally, except for a few examples, psychosocial needs were mainly limited to encompass social factors, especially in relation to loneliness. One may question the single-sided focus on social needs, especially because unrest, anxiety and depression are common symptoms in home-dwelling PWDs [12].

HCPs perceiving fulfilling psychosocial needs as a natural and integrated part of their practice will have a different approach to psychosocial needs than HCPs viewing fulfilment of psychosocial needs as a separate task [30]. If meeting psychosocial needs are viewed as separate tasks and are additionally not mentioned in the administrative decisions, this may decrease the fulfilment of these needs. However, interventions to meet psychosocial needs, like comforting an uneasy PWD, can be difficult to describe in an administrative decision. What the HCPs perceive as psychosocial needs and interventions for meeting these are essential for the fulfilment of these needs [30]. A broad view that considers smaller “interventions” for meeting psychosocial needs, such as having a short conversation, may increase the HCPs perception of responsibility for meeting these needs. Likewise, if interventions are only perceived as “larger” interventions, like a day care centre, the responsibility for meeting psychosocial needs may decrease. More comprehensive or time-demanding interventions cannot be seen within the HCPs’ role to fulfil. Furthermore, the home care services cannot be expected to fulfil all PWDs’ needs, a limit for the care provided must exist [33]. However, when HCPs observe needs they are not able to meet, they should take the role of being a link to achieve contact between PWDs, their relatives, other healthcare services or volunteer organizations [27]. As this study indicates, this role was fulfilled to a greater extent by contacting family, but rarely voluntary organizations. This finding must be viewed in accordance with the HCPs’ limited knowledge concerning voluntary organizations and the lack of structured cooperation between home care services and voluntary organizations.

Knowledge of the PWD, a trusting relationship, and individual care provided by a limited number of known HCPs with sufficient competence and time are described as essential in dementia care [2, 28, 30]. Providing individual, person-centred care, may be perceived as too time consuming, especially if the goal for the care provision is to provide care strictly in accordance with limited allocated time. Providing generalized and standardized care may be viewed as more time-effective [6, 56]. On the other hand, using time to get to know the PWD and provide individual and flexible care in which, psychosocial needs are also emphasized can be perceived as time-saving [57]. This view was especially evident in the integrated logic. In particular, by using time to get to know the PWD and establish a trusting relationship, care could be easier to conduct [57]. Furthermore, utilizing opportunities and searching for freedom to operate flexibly, within but also beyond the timeframe, seems to enable HCPs to meet PWDs’ psychosocial needs in a more adjusted way [33, 58]. When HCPs experience that they have insufficient competence and time, which was particularly prominent in the renouncement logic, the HCPs may feel forced to adjust care to the available recourses and not in accordance with the actual needs of the PWDs. Moreover, the HCPs adjustment may conflict with their professional standards and ethics and create frustration [31, 33]. HCPs are situated in a continually challenging situation where they are determined to fill in an impossible gap between what is perceived as good quality care and the resources available for providing it [31, 33].

Despite health authorities request for holistic care [2, 5], the HCPs in our study described psychosocial needs to rarely be mentioned in administrative decisions. How HCPs perceived administrative decisions and how strictly they followed them may influence the provided care [41]. That psychosocial needs seldom were mentioned in the administrative decisions seemed to especially affect care provided by HCPs who operated strictly in accordance with the administrative decisions, as in the physical need-oriented logic. That HCPs adjusted care to fit the administrative decisions can be seen in relation to taking responsibility for the municipality’s requirements for efficiency and for providing equal service to all persons receiving home care services in their district [41]. On the other hand, one might argue that by delivering care strictly in accordance with administrative decisions, the HCPs’ loyalty to the municipality’s system is seen as more important than the actual needs of the PWD and the loyalty to the PWD. A home care service that focuses on organizationally driven care is in risk of decreasing care that is flexible and tailored to the individual PWD’s actual needs [41]. Furthermore, the interaction between the HCPs and the PWD may become impersonal and decrease the PWD ability to promote his/hers opinion and views [27]. However, setting aside administrative decisions may result in delayed or reduced care for others. On the other hand, providing care beyond the administrative decisions can be seen as a strong obligation to provide quality care in accordance with professional and ethical norms, for instance by prioritizing sitting down to talk with an anxious PWD. By practicing professional autonomy with an emphasis on quality care according to norms and values, the HCPs may be better enabled to conduct assessments and provide care in which the individual PWD’s specific situation and needs are addressed [41, 59].

Another interesting finding in this study is that the three different logics were found at the same work place and that they seemed to co-exist in all four municipalities. In the literature, co-existing, often competing, logics have been described [59, 60], as for instance the logic of performance management and the logic of profession [60]. As the three logics described in the present study differed in how they perceived their role related to PWDs’ psychosocial needs, is it relevant to question how and why these logics could co-exist within the same workplace. One explanation may be that the provision of care seemed to be influenced not only by contextual factors, such as equal allocated time to provide care, but also by individual factors [30, 61], as nurses and assistant nurses from all four municipalities were represented in all three logics. Subjective views and perceptions may also play an important role in why HCPs in one logic could set aside administrative decisions to provide individual and extended care, while others choose to pay less attention to individual PWDs needs in favour of following administrative decisions [41]. Another reason for the co-existence of the three logics may be that the home care service, as it is organized, actually depends both on those who follow the administrative decisions and those who go further to fulfil the municipalities’ responsibilities and duties. It may also be that the co-existing logics are appropriate and desirable viewed from a management perspective. Despite that the logics in this study apparently seemed to operate well beside each other, it would be expedient to further explore such logics impact on each other and the possible competing interests between them.

In a context where home care services report tight financial constraints with a high focus on efficiency, providing high quality services that emphasizes individual service beyond physical needs seems to be a great challenge [11, 33]. Some voices have advocated that the healthcare services must look for new or other ways of organizing and delivering services to be able to offer good dementia care [42]. However, to be able to meet psychosocial needs among PWDs, central governments are emphasizing collaboration between HCPs, PWDs, relatives and voluntary organizations [5]. According to the WHO [1], the complexity of dementia care, which involves health and social care, the family, and the private and voluntary sectors, poses a barrier towards prioritization and obscures who should take responsibility [1]. Better cooperation between the different actors who provide care [62] and a debate concerning the extent to which and from whom PWDs should receive help fulfilling psychosocial needs are needed. HCPs in home care services are providing care to a number of persons with different ages, diagnoses and care needs. Thus, ensuring that HCPs have sufficient competence to understand the particular situation of the PWDs needs for assistance related to psychosocial needs is a significant issue, especially since PWDs are often in a vulnerable situation, unable to safeguard their psychosocial needs on their own. Within home care services, HCPs cannot be expected to take full responsibility for meeting the psychosocial needs of PWDs. However, if HCPs are not meeting the psychosocial needs of PWDs, at least to some degree, one might question if such a situation can threaten the political aim that states that PWDs shall be enabled to stay in their own homes as long as possible while receiving high quality holistic care.

### Strengths and limitations

One strength of this study is that the same author conducted all focus group interviews and transcribed them. Another strength is that the data were discussed thoroughly among the three authors, providing a broader and more nuanced understanding. One limitation is that only female HCPs participated in the study, which may have influenced the findings. However, the home care services are mainly staffed by women. Another limitation of this study is that the findings cannot be generalized however, this study identified elements that may have relevance for home care services in other parts of Norway or other countries. The present study investigated HCPs’ perceptions of psychosocial needs, which could be a strength if future research includes observational studies and also includes PWDs’ and relatives’ experiences regarding the assessment and fulfilment of the psychosocial needs of PWDs, and their families in homecare settings.

## Conclusions

The differences in how health care providers perceived the psychosocial needs of PWDs and their responsibilities for meeting those needs influenced how the psychosocial needs of PWDs were met.

Our study suggests that the differences in the HCPs’ perceptions related to psychosocial needs of PWDs indicates a need for a clarification of the concept and a discussion of who should be responsible for meeting these needs. Further, increased competence and consciousness regarding psychosocial needs and how to fulfil these needs are essential for delivering holistic care that enables PWDs to live in their own homes for as long as possible. These suggestions may be of interest to policy-makers, leaders and HCPs in home care services who are seeking to enhance these services to better meet the psychosocial needs of older home-dwelling PWDs.

## Additional file

Additional file 1: Interview guide. An interview guide developed for the present study, translated from Norwegian. (DOCX 13 kb)

## Abbreviations

HCP(s): Health care provider(s)
PWD(s): Person(s) with dementia
WHO: World Health Organisation
