# Supplementary material for: Meeting psychosocial needs for persons with dementia in home care services – a qualitative study of different perceptions and practices among health care providers
Source: BMC Geriatr. 2017 Sep 11;17:211. doi: 10.1186/s12877-017-0612-3 (PMC5594550; doi:10.1186/s12877-017-0612-3)
Supplement: Additional file 1: — Interview guide. An interview guide developed for the present study, translated from Norwegian. (DOCX 13 kb) [file 12877_2017_612_MOESM1_ESM.docx]

**Interview guide**

| **Question** |  |
| --- | --- |
| **1** | Regarding the administrative decisions. Do you always read them? What about any possible additional documents made by the purchasers, do you have access to those? |
| **2** | In what way is psychosocial health and needs described in the administrative decisions? |
| **3** | How do you understand psychosocial needs? |
| **4** | What do you do to identify and assess psychosocial needs and how are observed needs handled? |
| **5** | How do you meet PWDs’ psychosocial needs? |
| **6** | How do the administrative decisions influence on your professional judgement and the services you provide? |
| **7** | What do you do if you discover needs that are not described in the administrative decision? |
| **8** | Can you please describe how you perceive the correlation between the administrative decisions and the services you actually provide? |
| **9** | Can you please describe how observations related to psychosocial health and needs are documented? |
| **10** | Do you perceive psychosocial needs as necessary basic needs that are within the municipalities’ responsibility to fulfil? Why/why not? |
| **11** | Do you know any governing documents or guidelines that describe the municipalities’ responsibility to safeguard psychosocial health? |
| **12** | Could you please describe the ideal service that would optimally meet the psychosocial needs of the PWDs? |
